# A retrospective global study of the prevalence of O-serotypes of invasive *Escherichia coli* disease in patients admitted to tertiary care hospitals

**DOI:** 10.1017/S0950268825100344

**Published:** 2025-09-03

**Authors:** Jeroen Geurtsen, Joachim Doua, Luis Martinez-Martinez, Patricia Ibarra de Palacios, Jeff Powis, Matthew Sims, Peter Hermans, Olivier Barraud, Philippe Lanotte, Joshua Thaden, Oscar Go, Bart Spiessens, Darren Abbanat, Florian Wagenlehner, Tetsuya Matsumoto, Marc Bonten, Michal Sarnecki, Jan Poolman

**Affiliations:** 1 Bacterial Vaccines Discovery and Early Development, Janssen Vaccines & Prevention B.V., Leiden, The Netherlands; 2 Janssen Research & Development, Infectious Diseases & Vaccines, Janssen Pharmaceutica, Beerse, Belgium at the time of analysis; 3 Microbiology Unit, University Hospital Reina Sofia, Córdoba, Spain; 4 Department of Agricultural Chemistry, Soil Science and Microbiology, University of Cordoba, Maimonides Biomedical Research Institute of Cordoba (IMIBIC), Córdoba, Spain; 5 Centro de Investigación Biomédica en Red de Enfermedades Infecciosas (CIBERINFEC), Instituto de Salud Carlos III, Madrid, Spain; 6 Clinical Development, Janssen Research & Development, Bern, Switzerland at the time of analysis; 7 Department of Medicine, University of Toronto, Toronto, ON, Canada; 8 Department of Infection Prevention and Control, Michael Garron Hospital, Toronto, ON, Canada; 9 Department Internal Medicine, Section of Infectious Diseases and International Medicine, Corewell Health William Beaumont University Hospital, Royal Oak, MI, USA; 10 Departments of Internal Medicine & Foundational Medical Studies, Oakland University William Beaumont School of Medicine, Rochester, MI, USA; 11 Julius Center for Health Sciences and Primary Care, University Medical Center, Utrecht, The Netherlands; 12 European Clinical Research Alliance on Infectious Diseases, Utrecht, The Netherlands; 13 Department of Bacteriology, CHU Limoges, Limoges, France; 14 Department of Bacteriology and Department of Microbiology, Bretonneau Hospital Tours University, University of Tours-INRAE, Tours, France; 15 Department of Medicine, Duke University School of Medicine, Durham, NC, USA; 16 Janssen Research & Development, Raritan, NJ, USA; 17 Janssen Research & Development, Raritan, NJ, USA at the time of analysis; 18 Department of Urology, Pediatric Urology and Andrology, Justus-Liebig University, Giessen, Germany; 19 Department of Infectious Diseases, International University of Health and Welfare, Chiba, Japan; 20 Clinical Development, Janssen Vaccines, Bern, Switzerland at the time of analysis

**Keywords:** *E. coli*, EXPEC9V, O-serotype, invasive *E. coli* disease, vaccine

## Abstract

Invasive *Escherichia coli* disease (IED) is associated with high hospitalization and mortality rates, particularly among adults aged ≥60 years. O-antigens are virulence factors required for *E. coli* survival. To inform EXPEC9V development, a novel glycoconjugate vaccine targeting *E. coli* O-antigens that is no longer in active clinical development, this retrospective observational study describes O-serotype prevalence among *E. coli* isolates from IED patients. Eligible patients were identified from medical record databases (9 January 2018–8 November 2019) across 17 tertiary care hospitals in Europe, North America, and Asia. To estimate vaccine serotype coverage of EXPEC9V, *E. coli* isolates were O-serotyped using whole-genome sequencing and agglutination. Antimicrobial susceptibility testing was also performed. Nine hundred and two patients were enrolled, of whom 690 (76.5%) were aged ≥60 years. Common serotypes were O25, O2, O6, O1, O15, O75, O16, O4, and O18, with O25 being the most reported (17.3%). In patients aged ≥60 years, 422/637 *E. coli* isolates were EXPEC9V O-serotypes. EXPEC9V O-serotype prevalence did not substantially differ when stratified according to sex, presence of a positive blood culture, sepsis, fatality, or multidrug resistance. Consistent with previous studies, serotype O25 was most prevalent and associated with ~20% of cases. An EXPEC9V vaccine serotype coverage of 66.2% was observed for IED patients aged ≥60 years.

## Key results

- O25 was the most prevalent O-serotype among *E. coli* isolates, consistent with previous studies
- 422/637 *E. coli* isolates from patients aged ≥60 years were EXPEC9V O-serotypes, corresponding to a vaccine serotype coverage of 66.2% in this high-risk population, although this vaccine is no longer in active clinical development
- EXPEC9V O-serotype prevalence did not substantially differ when stratified according to sex, presence of a positive blood culture, sepsis, fatality, or multidrug resistance; this suggests EXPEC9V serotype coverage would not substantially differ among these subpopulations

## Introduction

Extraintestinal pathogenic *E. coli* (ExPEC) is an important *E. coli* pathogroup capable of causing infections outside the gastrointestinal tract [1]. Besides being the most common cause of urinary tract infections (UTIs), ExPEC is the leading – yet under-recognized – cause of bacteraemia in adults worldwide, as well as a major cause of community-onset sepsis and subsequent hospitalization, and is associated with high morbidity and mortality [2–6]. Invasive *E. coli* disease (IED), which includes *E. coli* bacteraemia and sepsis, can be clinically defined as an *E. coli* infection with acute systemic consequences, which is microbiologically confirmed either by the isolation and identification of *E. coli* from the blood or in any other normally sterile body site; or by the isolation and identification of *E. coli* from the urine of patients with urosepsis and no other identifiable source of infection apart from the urinary tract [1, 5, 6].

In the US, data from 2003 suggested that up to 40,000 patient deaths are caused by *E. coli* sepsis each year. Assuming mortality increased in line with septicaemia-related hospitalizations, this figure is estimated to have increased to >85,000 deaths in 2014 [5, 7]. In a cohort study of 17,430 US patients with culture-positive community-onset sepsis, *E. coli* was identified as the most common causative pathogen, responsible for 33.7% of community-onset sepsis cases [6]. A more recent systematic study on the global burden of bacterial antimicrobial resistance demonstrated that sepsis-related mortality rates sharply increased in 2020/2021 and, by 2019, the likelihood that these deaths were the result of drug-resistant infections was 33% higher than in 1990 (8.5% vs. 6.4%) [8].

ExPEC strains possess a broad range of virulence traits and exhibit considerable genomic diversity [1]. O-antigens are important surface-exposed virulence factors necessary for pathogen survival, which makes them attractive targets for vaccine development. However, to date, over 180 different *E. coli* O-serotypes have been described representing a broad structural diversity [9, 10]. Yet, recent studies suggest that for IED, like for invasive pneumococcal disease, the majority of disease is associated with a specific subset of serotypes, with O25, O6, O1, and O2 among the most frequently associated, which considerably narrows down the number of O-serotypes that would be relevant for vaccine development [11–17].

Given the high incidence of IED, especially in adults aged 60 years and older, with an associated case-fatality rate of >10%, prevention of IED, including *E. coli* bacteraemia and sepsis, has been identified as an important unmet medical need [7, 18]. Despite available treatment options, the clinical and economic impact of IED remains substantial and novel preventive strategies, including vaccines, are needed [7, 18, 19].

Based on the overall prevalence of O-serotypes among *E. coli* isolated from patients with IED, EXPEC9V – a 9-valent bioconjugate vaccine targeting nine *E. coli* serotypes (O1A, O2, O4, O6A, O15, O16, O18A, O25B, and O75) – was developed and was being evaluated in a pivotal phase 3 vaccine efficacy trial (E.mbrace; NCT04899336) for the prevention of IED in adults ≥60 years with a recent history of UTI at the time of study conduct, although it is no longer in active clinical development [20].

To inform the development of EXPEC9V, this study describes the prevalence of O-serotypes of *E. coli* isolated from hospitalized patients with IED in 17 tertiary care hospitals across eight different countries. We report the serotype coverage of the EXPEC9V vaccine among patients with IED, with a focus on those aged 60 years and older. To estimate vaccine coverage in subgroups of interest, results were stratified by sex, the absence or presence of a positive blood culture, sepsis, a fatal outcome, and multidrug resistance.

## Methods

### Study design and participants

This retrospective, multicentre, non-interventional study analysed data collected across 17 tertiary care hospitals in Europe, North America, and Asia. IED isolates collected from eligible patients across sites in Canada (2), France (2), Germany (2), Italy (2), Japan (2), Spain (3), the United Kingdom (2), and the United States (2) were included in the analysis [21]. Sites were selected based on their ability to access demographic and clinical data of eligible patients retrospectively. The detailed study methods and primary results – which describe the clinical profile of IED in this cohort of patients and the utility of a proposed IED case definition – have been published previously [21].

The study was initiated on 28 September 2018 and included data collected between 09 January 2018–08 November 2019. The primary data sources for this study were the medical records of each participating patient from the study site (i.e., any clinical records – including microbiology laboratory records – and hospital records) [21].

Patients with IED of any age were identified from these medical record databases using a list of International Classification of Diseases (ICD) codes, which included culture-confirmed bacteraemia and sepsis. Patient records found to include the relevant ICD codes during the 12 months prior to the start of the study were assessed for eligibility as described previously [21]. Patients were included in the study if they had been hospitalized for IED or had nosocomial IED, with either *E. coli* as a single pathogen or a mix of pathogens where *E. coli* was present. For this, the *E. coli* infection had to be laboratory confirmed by culture, with the patient showing signs and symptoms of invasive disease based on the presence of systemic inflammatory response syndrome (SIRS), sepsis, or septic shock.

### Patient consent

This study was approved by the Independent Ethics Committee and/or Institutional Review Boards. Physicians sought waivers and/or consent from eligible patients for inclusion of their data into the study according to local regulations. A waiver for informed consent was obtained for Canada, the United Kingdom, and the United States. In Germany, all patients signed a participation agreement/informed consent form (ICF)/informed assent form (IAF); and for deceased patients, a participation agreement/ICF/IAF was signed by the patient’s next of kin. In Spain and Italy, attempts to contact the patients were made, but waivers were obtained if it was too difficult to contact the patient. In France, letters of nonobjection were sent to the patients, which explained that the patients were included without consent if no objection was made. In Spain, France, and Italy, no consent was required for deceased patients. In Japan, no consent was required, but patients were given an opportunity to refuse study participation [21].

### Species identification and antimicrobial susceptibility testing

Suspected *E. coli* strains isolated from patients with IED were shipped to a certified central laboratory using Stuart Transport Medium at ambient temperature. The shipped strains were checked for purity by culture, and *E. coli* species identity was confirmed by matrix-assisted laser desorption ionization time-of-flight (MALDI-TOF) mass spectrometry. Automated susceptibility testing was performed using the BD Phoenix™ system with NMIC-417 or NMIC-408 panels (depending on the strain), according to the broth microdilution assay as per Clinical and Laboratory Standards Institute (CLSI) guidelines (M07 supplement) [22], with interpretations regarding susceptibility or resistance reported according to European Committee on Antimicrobial Susceptibility Testing (EUCAST) established breakpoints, as appropriate [22, 23]. Resistance to antibiotics of the following classes was tested at a minimum: aminoglycoside, fluoroquinolone, folate pathway inhibitor, nitrofurantoin, and β-lactam. For a full list of antibiotics included in susceptibility testing, see Supplementary Table S1. Multidrug resistance was defined as resistance to at least three antimicrobial drug classes.

### Determining O-serotypes

All *E. coli* isolates were analysed by agglutination O-serotyping [24]. Whole-genome sequencing (WGS) was performed using Illumina® technologies, and the identification of O-serotypes was achieved using unique O serotype-specific sequences of *wzy*, *wzx*, *wzt*, and *wzm* genes following guidelines and using reference sequences described elsewhere [9, 10, 17]. To estimate the serotype coverage of EXPEC9V, the cumulative prevalence of the following nine O-serotypes (based on agglutination or WGS) was reported: O1, O2, O4, O6, O15, O16, O18, O25, and O75. *E. coli* isolates for which the O-serotype could not be determined were excluded from the analyses. Further subgroup analyses were performed to assess O-serotype prevalence among isolates from patients stratified according to age (all patients with IED; patients aged ≥60 years), sex, whether they had a positive *E. coli* blood culture, sepsis, a fatal outcome, or a multidrug-resistant (MDR) infection or not.

### Statistical analysis

Comparisons were summarized using descriptive statistics (mean, standard deviation (SD), maximum, upper quartile, median, lower quartile, interquartile range, minimum, and number of observations, as appropriate) for continuous parameters, and frequency and proportions for categorical parameters. No formal statistical hypothesis testing was conducted for this study. All 95% confidence intervals (CIs) were two-sided.

## Results

### IED was diagnosed across different ethnic groups, equally affected both males and females, and occurred most frequently in older adults

A total of 924 patients were screened, of whom 902 patients were included in the study. Reasons for screening failure included: absence of culture confirmation (10 patients); no IED diagnosis within the last 12 months (five patients); no hospitalization for IED or not being in the hospital at the time of IED occurrence (four patients); multiple inclusion criteria not met (two patients); and inability to acquire informed consent (one patient). A total of 898 patients (99.6%) completed the study; reasons for discontinuation included: *E. coli* isolate was lost (two patients); *E. coli* isolate was not sent (one patient); and *E. coli* isolate was not frozen (one patient).

Baseline characteristics and the clinical profile of patients enrolled in this study have previously been published [21], and are summarized in Table 1. Briefly, the median age of the study population was 71 (range, 61–82) years; 465 patients (51.6%) were male, and 690 (76.5%) patients were aged ≥60 years. Most patients had IED with a positive *E. coli* blood culture (*n* = 844 [93.6%]), while 326 (36.1%) patients showed signs or symptoms of a UTI. 698 (77.4%), 588 (65.3%), and 127 (14.1%) patients presented with SIRS, sepsis, and septic shock respectively. Overall, 180 (20.0%) patients died, of whom 171 had a cause of death recorded (52 [30.4%] related to IED; 84 [49.1%] related to underlying conditions; and 35 [20.5%] related to other causes) [21].

**Table 1.** Patient demographics and characteristics

Characteristic	All IED (*N* = 902)	Patients ≥60 years (*N* = 690)
Male, *n* (%)	465 (51.6)	373 (54.1)
Age, years, median (range^a^)	71 (61–82)	76 (69–84)
BMI, kg/m^2^, mean (SD)	26.0 (7.36)	25.9 (6.2)
Classification of IED, *n* (%)		
Bacteraemic	844 (93.6)	646 (93.6)
Nonbacteraemic	58 (6.4)	44 (6.4)
Signs and symptoms of IED at diagnosis, *n* (%)		
Nausea/vomiting	245 (27.2)	181 (26.2)
General symptoms (chills, malaise, fatigue, muscle pain)	412 (45.7)	323 (46.8)
Signs or symptoms of UTI	326 (36.1)	244 (35.4)
Altered mental state	154 (17.1)	140 (20.3)
Hypotension	290 (32.2)	224 (32.5)
Hypothermia	48 (5.3)	40 (5.8)
SIRS	698 (77.4)	525 (76.1)
Sepsis (physician’s assessment)	588 (65.2)	465 (67.4)
Septic shock (physician’s assessment)	127 (14.1)	105 (15.2)
Mortality	180 (20.0)	151 (21.9)

a Interquartile range. BMI, body mass index; IED, invasive *E. coli* disease; SIRS, systemic inflammatory response syndrome; UTI, urinary tract infection.

### O25 the most common O-serotype, consistent with previous epidemiological reports, and EXPEC9V had a vaccine coverage of around 66%

**Figure 1.**
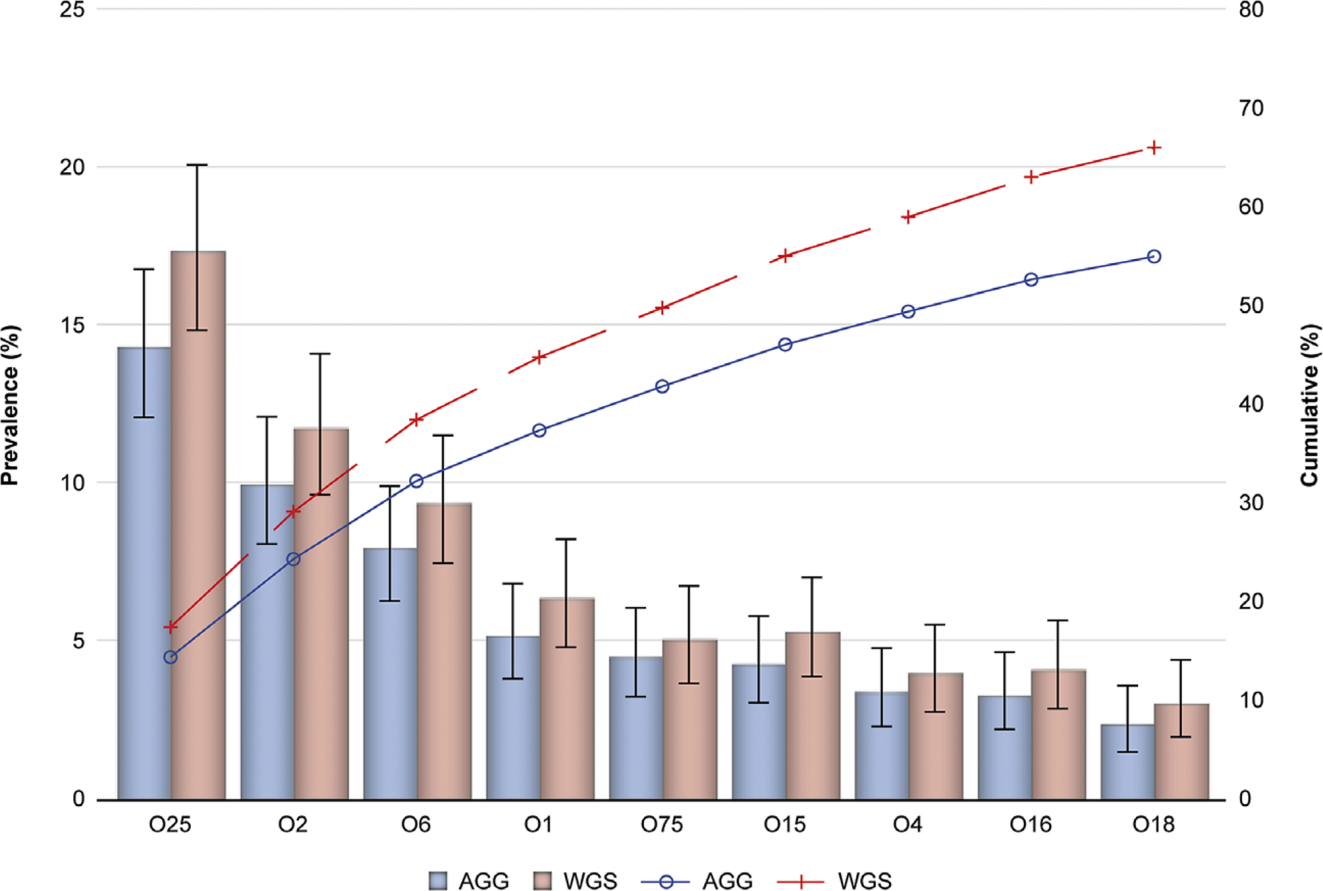
Prevalence (%) of EXPEC9V O-serotypes based on agglutination and whole-genome sequencing with cumulative prevalence (%) for all IED isolates; full analysis set. 95% confidence interval based on the exact Clopper–Pearson method. AGG, agglutination; IED, invasive *E. coli* disease; WGS, whole-genome sequencing.

**Figure 2.**
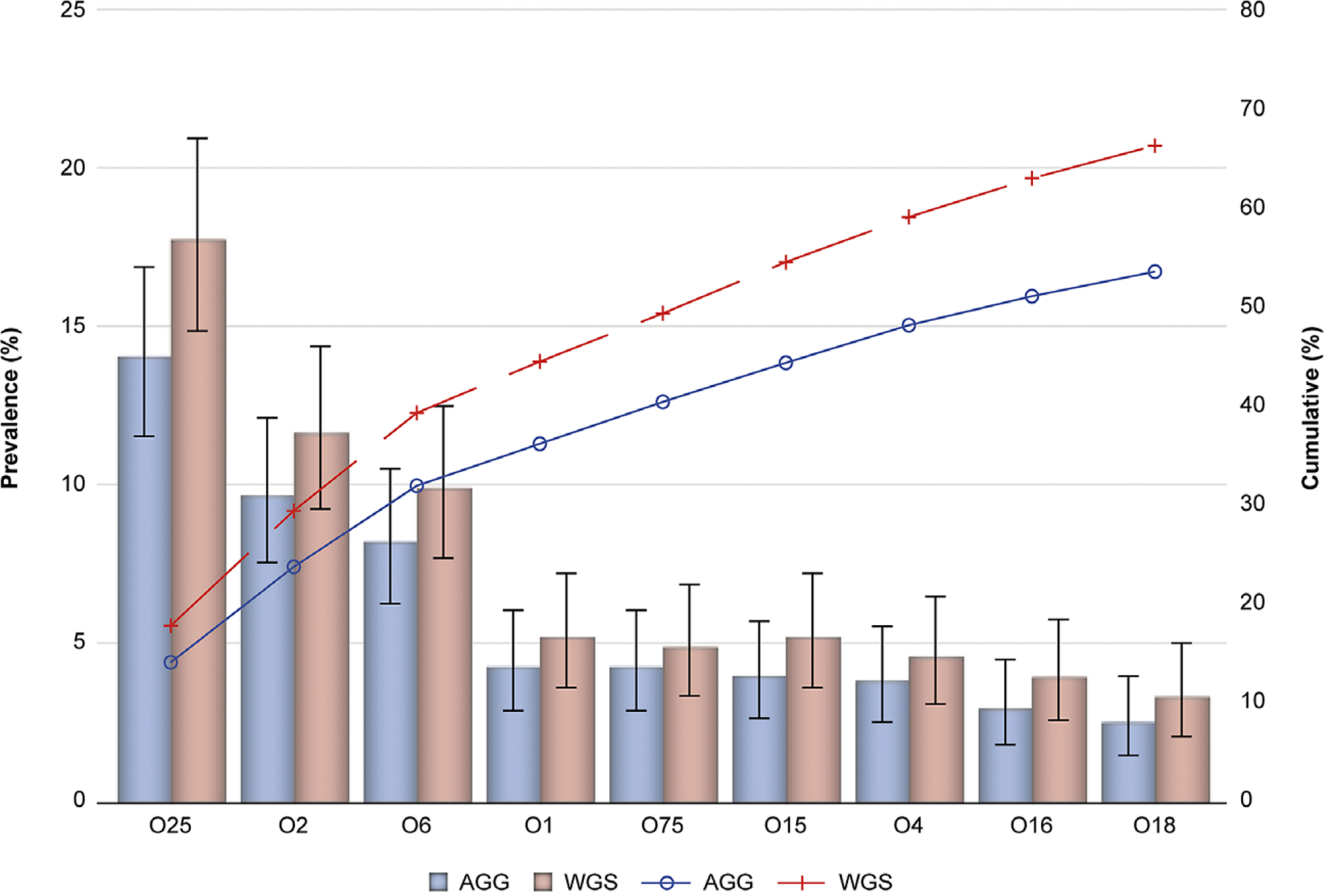
Prevalence (%) of EXPEC9V O-serotypes based on agglutination and whole-genome sequencing with cumulative prevalence (%) for IED isolates from patients aged ≥60 years old; full analysis set. 95% confidence interval based on the exact Clopper–Pearson method. AGG, agglutination; IED, invasive *E. coli* disease; WGS, whole-genome sequencing.

Out of 896 *E. coli* isolates that underwent WGS, O-serotype prevalence could be determined in 837 (93.4%) isolates, and 76 unique O-serotypes were identified. Among these, the most observed serotypes were O25 (17.3%), O2 (11.7%), O6 (9.3%), O1 (6.3%), O15 (5.3%), O75 (5.0%), O8 (4.2%), O16 (4.1%), O17/O44/O73/O77/O106 (4.1%), O4 (3.9%), O18 (3.0%), and O9 (2.7%) (Supplementary Table S2). Based on this WGS analysis, a total of 552/837 *E. coli* IED isolates were identified as EXPEC9V O-serotypes, corresponding to a vaccine serotype coverage of 66.0%. In patients aged ≥60 years, 422/637 IED isolates were identified as EXPEC9V O-serotypes, corresponding to a vaccine serotype coverage of 66.2%. The EXPEC9V vaccine had a cumulative serotype coverage of just over 60% among all isolates O-serotyped by WGS and of approximately 55% assessed by agglutination, with similar prevalence in all patients with IED and in patients aged ≥60 years (Figures 1 and 2).

After stratifying the prevalence of IED isolates among patients aged ≥60 years by those who presented with a positive *E. coli* blood culture (*n* = 595) and those who did not (*n* = 42), serotypes O25, O2, and O6 were among the most prevalent of the EXPEC9V serotypes in both subgroups: O25 (IED with a positive *E. coli* blood culture, 18.2% vs. IED without a positive *E. coli* blood culture, 11.9%), O2 (11.8% vs. 9.5% respectively), and O6 (10.1% vs. 7.1% respectively) (Table 2).

**Table 2.** Prevalence of EXPEC9V O-serotypes based on whole-genotype sequencing, stratified by the recording of a positive *E. coli* blood culture, in patients aged ≥60 years

	IED with a positive *E. coli* blood culture		IED without a positive *E. coli* blood culture	
O-serotype	Number of isolates (*N = 595*)	Prevalence (95% CI) 1.0000	Number of isolates (*N = 42*)	Prevalence (95% CI) 1.0000
EXPEC9V	401	0.6739 (0.6346, 0.7115)	21	0.5000 (0.3419, 0.6581)
O1	32	0.0538 (0.0371, 0.0751)	1	0.0238 (0.0006, 0.1257)
O2	70	0.1176 (0.0929, 0.1463)	4	0.0952 (0.0266, 0.2262)
O4	28	0.0471 (0.0315, 0.0673)	1	0.0238 (0.0006, 0.1257)
O6	60	0.1008 (0.0778, 0.1279)	3	0.0714 (0.0150, 0.1948)
O15	31	0.0521 (0.0357, 0.0731)	2	0.0476 (0.0058, 0.1616)
O16	23	0.0387 (0.0247, 0.0574)	2	0.0476 (0.0058, 0.1616)
O18	21	0.0353 (0.0220, 0.0534)	0	0 (0, 0.0841)
O25	108	0.1815 (0.1513, 0.2149)	5	0.1190 (0.0398, 0.2563)
O75	28	0.0471 (0.0315, 0.0673)	3	0.0714 (0.0150, 0.1948)
Other^a^	194	0.3261 (0.2885, 0.3654)	21	0.5000 (0.3419, 0.6581)

a Non-EXPEC9V O-serotypes.

*Note:* IED, invasive *E. coli* disease.

Subgroup analysis of EXPEC9V O-serotype prevalence among patients aged ≥60 years stratified by sex is presented in Table 3. O25 was the most prevalent O-serotype in both females (17.7%) and males (17.8%). Notably, serotype O1 was more common in IED isolates from females compared to males (8.3% vs. 2.6% respectively), as was serotype O16 (6.3% vs. 2.0% respectively). Overall, the prevalence of EXPEC9V IED isolates among female and male patients with IED aged ≥60 years did not substantially differ (70.8% and 62.5% respectively).

**Table 3.** Prevalence of EXPEC9V O-serotypes based on whole-genotype sequencing, stratified by sex, in patients aged ≥60 years

	Female		Male	
O-serotype	Number of isolates (*N = 288*)	Prevalence (95% CI) 1.0000	Number of isolates (*N = 349*)	Prevalence (95% CI) 1.0000
EXPEC9V	204	0.7083 (0.6521, 0.7602)	218	0.6246 (0.5715, 0.6756)
O1	24	0.0833 (0.0541, 0.1215)	9	0.0258 (0.0119, 0.0484)
O2	38	0.1319 (0.0951, 0.1766)	36	0.1032 (0.0733, 0.1399)
O4	11	0.0382 (0.0192, 0.0673)	18	0.0516 (0.0309, 0.0803)
O6	26	0.0903 (0.0598, 0.1295)	37	0.1060 (0.0758, 0.1432)
O15	13	0.0451 (0.0243, 0.0760)	20	0.0573 (0.0354, 0.0871)
O16	18	0.0625 (0.0375, 0.0970)	7	0.0201 (0.0081, 0.0409)
O18	11	0.0382 (0.0192, 0.0673)	10	0.0287 (0.0138, 0.0521)
O25	51	0.1771 (0.1348, 0.2262)	62	0.1777 (0.1390, 0.2219)
O75	12	0.0417 (0.0217, 0.0717)	19	0.0544 (0.0331, 0.0837)
Other^a^	84	0.2917 (0.2398, 0.3479)	131	0.3754 (0.3244, 0.4285)

a Non-EXPEC9V O-serotypes.

### Resistance to ≥1 drug and ≥1 drug class was common, at around 66% of isolates

In the total study population, 587 out of 895 *E. coli* isolates (65.6%) tested for susceptibility were found to be resistant to ≥1 antibiotic in ≥1 drug class, with >25% of the isolates being resistant to amoxicillin, piperacillin, amoxicillin/clavulanate, trimethoprim/sulfamethoxazole, ciprofloxacin, and levofloxacin (Supplementary Table S1). Among patients aged ≥60 years with IED, 450 (65.9%) *E. coli* isolates were found to be resistant to ≥1 drug class, with 134 (19.6%) considered as MDR (Table 4). The prevalence of EXPEC9V O-serotypes among MDR *E. coli* isolates from patients with IED aged ≥60 years is presented in Supplementary Figure S1, indicating a cumulative prevalence of approximately 65% among MDR isolates O-serotyped by WGS, which is similar to the vaccine serotype coverage of EXPEC9V in the total study population and patients aged ≥60 years (Figures 1 and 2).

**Table 4.** Antibiotic resistance in patients aged ≥60 years

	Number of isolates (*N* = 683)^a^
Resistance, *n* (%)	
Susceptible^b^	233 (34.1)
MDR^c^	134 (19.6)
Resistance to single antibiotics, *n* (%)	
Aminoglycoside	
Amikacin	2 (0.3)
Gentamicin	62 (9.1)
Tobramycin	73 (10.7)
Fluoroquinolone	
Ciprofloxacin	185 (27.1)
Levofloxacin	175 (25.6)
Folate pathway inhibitors	
Trimethoprim	121 (17.7)
Trimethoprim/sulfamethoxazole	192 (28.1)
Nitrofurantoin	2 (0.3)
β-lactam	
Amoxicillin	395 (57.8)
Amoxicillin/clavulanate	227 (33.2)
Aztreonam	54 (7.9)
Cefepime	75 (11.0)
Cefoxitin	38 (5.6)
Ceftazidime	76 (11.1)
Ceftriaxone	107 (15.7)
Cefuroxime	145 (21.2)
Ertapenem	3 (0.4)
Imipenem	1 (0.1)
Meropenem	1 (0.1)
Piperacillin	369 (54.0)
Piperacillin/tazobactam	26 (3.8)
Temocillin	56 (8.2)

*Note:* Susceptibility results are based on EUCAST 2022 breakpoints. EUCAST, European Committee on Antimicrobial Susceptibility Testing; IED, invasive *E. coli* disease; MDR, multidrug resistant.

a 683/690 *E. coli* isolates from patients aged ≥60 years in the full analysis set underwent antibacterial susceptibility testing.

b Susceptible was based on susceptibility to representative antibiotics in the following five classes of antimicrobial drugs: aminoglycoside, fluoroquinolone, folate pathway inhibitor, nitrofurantoin, and β-lactam. Susceptibility was defined as the absence of resistance to 5/5 antimicrobial drug classes.

c MDR was based on resistance to representative antibiotics in the following five classes of antimicrobial drugs: aminoglycoside, fluoroquinolone, folate pathway inhibitor, nitrofurantoin, and β-lactam. MDR was defined as resistance to at least 3/5 antimicrobial drug classes.

### EXPEC9V O-serotype prevalence was similar irrespective of survival and sepsis status

From the total amount of IED isolates for which the O-serotype could be determined (*n* = 837), 165 (19.7%) were from patients who died, while 672 (80.3%) were from patients who survived for >28 days. The prevalence of EXPEC9V O-serotypes by survival status for patients aged ≥60 years with IED is shown in Table 5. Overall, the prevalence of EXPEC9V O-serotypes was similar among patients regardless of 28-day survival status (61.1% among patients with IED who died and 65.8% among patients with IED who survived); however, the prevalence of individual EXPEC9V O-serotypes varied between the two groups. Serotypes O1, O2, and O6 were more prevalent in IED isolates from patients alive after the 28-day follow-up period, while O25 was more prevalent among patients who had died. Similar variations in prevalence rates were observed among all patients with IED (Supplementary Table S3).

**Table 5.** Prevalence of EXPEC9V O-serotypes based on whole-genotype sequencing, stratified by mortality status, in patients aged ≥60 years

	Mortality: Yes		Mortality: No	
O-serotype	Number of isolates (*N* = 131)	Prevalence (95% CI) 1.0000	Number of isolates (*N* = 439)	Prevalence (95% CI) 1.0000
EXPEC9V	80	0.6107 (0.5216, 0.6946)	289	0.6583 (0.6119, 0.7026)
O1	2	0.0153 (0.0019, 0.0541)	24	0.0547 (0.0353, 0.0803)
O2	9	0.0687 (0.0319, 0.1264)	58	0.1321 (0.1019, 0.1674)
O4	5	0.0382 (0.0125, 0.0868)	22	0.0501 (0.0317, 0.0749)
O6	6	0.0458 (0.0170, 0.0970)	47	0.1071 (0.0797, 0.1398)
O15	10	0.0763 (0.0372, 0.1359)	20	0.0456 (0.0280, 0.0695)
O16	4	0.0305 (0.0084, 0.0763)	19	0.0433 (0.0263, 0.0668)
O18	5	0.0382 (0.0125, 0.0868)	11	0.0251 (0.0126, 0.0444)
O25	30	0.2290 (0.1602, 0.3105)	69	0.1572 (0.1244, 0.1947)
O75	9	0.0687 (0.0319, 0.1264)	19	0.0433 (0.0263, 0.0668)
Other^a^	51	0.3893 (0.3054, 0.4784)	150	0.3417 (0.2974, 0.3881)

a Non-EXPEC9V O-serotypes.

Four hundred and sixty-five (67.4%) patients with IED aged ≥60 years were diagnosed with sepsis by their physician, and 105 (15.2%) experienced septic shock (Table 1). Among the patients for whom the O-serotypes could be determined, the prevalence of EXPEC9V O-serotypes stratified by sepsis status is shown in Table 6. Overall, the prevalence of EXPEC9V O-serotypes was comparable among patients with and without sepsis (65.7% and 67.8% respectively), as was the overall prevalence of individual EXPEC9V O-serotypes. The prevalence of EXPEC9V O-serotypes by sepsis status for all patients with IED was also similar and is shown in Supplementary Table S4.

**Table 6.** Prevalence of EXPEC9V O-serotypes based on whole-genotype sequencing, stratified by sepsis status, in patients aged ≥60 years

	Sepsis: Yes		Sepsis: No	
O-serotype	Number of isolates (*N = 434*)	Prevalence (95% CI) 1.0000	Number of isolates (*N = 202*)	Prevalence (95% CI) 1.0000
EXPEC9V	285	0.6567 (0.6099, 0.7013)	137	0.6782 (0.6090, 0.7421)
O1	16	0.0369 (0.0212, 0.0592)	17	0.0842 (0.0498, 0.1313)
O2	53	0.1221 (0.0928, 0.1567)	21	0.1040 (0.0655, 0.1545)
O4	17	0.0392 (0.0230, 0.0620)	12	0.0594 (0.0311, 0.1015)
O6	38	0.0876 (0.0627, 0.1182)	25	0.1238 (0.0817, 0.1773)
O15	20	0.0461 (0.0284, 0.0703)	13	0.0644 (0.0347, 0.1075)
O16	18	0.0415 (0.0248, 0.0648)	7	0.0347 (0.0140, 0.0701)
O18	16	0.0369 (0.0212, 0.0592)	5	0.0248 (0.0081, 0.0568)
O25	85	0.1959 (0.1595, 0.2364)	28	0.1386 (0.0941, 0.1941)
O75	22	0.0507 (0.0320, 0.0757)	9	0.0446 (0.0206, 0.0829)
Other^a^	149	0.3433 (0.2987, 0.3901)	65	0.3218 (0.2579, 0.3910)

a Non-EXPEC9V O-serotypes.

## Discussion

Despite the key role that *E. coli* has in causing bacterial invasive disease – being the number one pathogen globally – there is a lack of national, regional, and global surveillance programmes to provide insight into disease incidence as well as associated O-serotype prevalence, in particular. This retrospective study describes O-serotype prevalence in general and of the now-discontinued EXPEC9V vaccine serotype coverage across a multinational cohort of patients hospitalized with IED. Serotypes O25, O1, O2, O6, O15, and O75 were identified as the most common across the IED isolates, with serotype O25 alone being reported for 17.3% of isolates from all patients with IED and 17.7% of isolates from patients ≥60 years old (based on WGS).

The observed overall O-serotype prevalence is consistent with previous multinational epidemiological reports. For example, the most common *E. coli* O-serotypes causing bloodstream infections in patients aged ≥60 years were identified as O25 (22.9%), O2, O6, O1, O75, O15, O8, O16, O4, O18, O77 group, O153, O9, O101/O162, O86, O13, O46/O134, and O83, all at a prevalence of ≥1% of isolates [17]. In another recent 10-year study by Lipworth et al. (2021), O-serotypes O1, O2, O4, O6, O8, O15, O16, O18, O25, and O75 made up 72% of 3,278 bloodstream *E. coli* isolates analysed in the United Kingdom [14]. Separately, studies in France and the Netherlands reported that the most common O-serotypes associated with bloodstream infections were O25, O8, O2, O6, and O15 [15, 16]. Furthermore, in a recent multicentric, prospective observational study among hospitalized patients with IED (*N* = 238), the analysis of blood and urine isolates demonstrated that O25, O6, and O1 were the most prevalent serotypes across three continents [25].

Previous studies have described how IED incidence increases and clinical prognosis, including risk of death, worsens with age [5, 26]. When comparing all patients with IED with patients aged ≥60 years in this study, a similar EXPEC9V vaccine serotype coverage was observed between the two groups (~66%). Overall, the prevalence of EXPEC9V O-serotypes among patients aged ≥60 years was found not to substantially differ when stratified by survival at 28-day follow-up or sepsis status. However, serotypes O1, O2, and O6 were more prevalent among patients who survived during the 28-day follow-up period, while O25 was more prevalent among those who had died. While this may be explained by O25 being associated with ST131 – a virulent *E. coli* clone – further investigations with increased numbers of isolates are needed to establish the link between specific O-serotypes and disease severity. When results were stratified by the absence or presence of a positive *E. coli* blood culture, O25, O2, and O6 were the most prevalent O-serotypes in both subgroups, although absolute prevalence rates varied. Yet, due to the low number of patients without a positive blood culture (*n* = 42), confidence intervals for these data are wide, and data should therefore be interpreted with care. The observed difference may be driven by potential selection bias as well as differential geographical representation.

*E. coli* serotype O25 was particularly prevalent among MDR isolates, reaching a proportion of almost 50%, which is consistent with the high prevalence of this O-serotype among MDR *E. coli* isolates in previous studies [14, 25]. The prevalence of MDR *E. coli* strains, such as those belonging to sequence type (ST) O25B:ST131, is increasing [25, 27], and the global emergence of MDR *E. coli* represents a major challenge for the prevention and management of *E. coli* infections that may often be lethal [17, 28].

### Strengths and limitations

A key strength of this study is that it was a multinational study across eight countries and three regions with relatively large sample sizes of clinical isolates linked to detailed clinical information. The results presented here align with other studies demonstrating the high prevalence of O25 and other IED-associated O-serotypes. This study also provides important real-world data on a pathogen that has historically been underappreciated in national healthcare systems.

As this was a retrospective, observational study, the quality of data was largely dependent on the availability and completeness of individual patients’ medical records. The retrospective nature of the study may have contributed to a low inclusion of patients where *E. coli* was only confirmed from a positive urine culture, as such isolates may not be routinely stored at hospital sites. The inability to randomly select study sites may have resulted in undetectable measurement errors from several sources, and systematic variation in how patients from different cultural backgrounds perceived illness and sought care.

While agglutination is considered the gold standard for O-serotyping, WGS is more versatile and less labour-intensive. Differences in O-serotype prevalence observed between the agglutination and WGS results may be related to the difference between phenotype and genotype, impacted by false-negative results, or O-antigen expression being absent in some *E. coli* isolates under in vitro testing conditions.

An important limitation is the relatively low number of sites and *E. coli* isolates analysed from each individual country, which hinders the ability to compare O-serotype prevalence and vaccine serotype coverage at a country level. Therefore, similar country-specific studies analysing larger numbers of IED isolates from a range of local sites are needed. Additionally, given the geography of participating sites and hospitals, which were predominantly located in high-income countries, further studies are needed to understand serotype coverage across low-to-middle-income countries. Finally, there was a lack of data on prior hospitalizations in other hospitals, which may have led to the misclassification of infections as community-acquired when they were healthcare-associated.

## Conclusion

In conclusion, there appears to be overall limited information on disease incidence and O-serotyping data for *E. coli* due to a lack of robust surveillance and disease awareness. Epidemiological data such as those presented here can help inform the development and adoption of novel preventative strategies, including vaccines. This study described the O-serotype prevalence and coverage of the EXPEC9V vaccine, which is no longer in active clinical development, and identified O25 as the most prevalent O-serotype among IED isolates. Overall, EXPEC9V vaccine serotype coverage was found to be approximately 66% independent whether this was analysed for all patients with IED, or patients aged ≥60 years. EXPEC9V O-serotype prevalence did not substantially differ when results were stratified according to whether patients aged ≥60 years had a positive *E. coli* blood culture, sepsis, survival at 28-day follow-up, as well as sex.

## Supporting information

10.1017/S0950268825100344.sm001 Geurtsen et al. supplementary material Geurtsen et al. supplementary material

## Data Availability

Janssen’s official data sharing statement (‘The data sharing policy of Janssen Pharmaceutical Companies of Johnson & Johnson is available at https://www.janssen.com/clinical-trials/transparency. As noted on this site, requests for access to the study data can be submitted through Yale Open Data Access [YODA] Project site at http://yoda.yale.edu’) will be provided to a journal/congress upon request.

## Supplementary material

The supplementary material for this article can be found at http://doi.org/10.1017/S0950268825100344.
