# Supplementary material for: A retrospective global study of the prevalence of O-serotypes of invasive Escherichia coli disease in patients admitted to tertiary care hospitals
Source: Epidemiol Infect. 2025 Sep 3;153:e109. doi: 10.1017/S0950268825100344 (PMC12529432; doi:10.1017/S0950268825100344)
Supplement: Geurtsen et al. supplementary material [file S0950268825100344sup001.docx]

*Epidemiology & Infection***A Retrospective Global Study of the Prevalence of O-serotypes of Invasive *Escherichia coli* Disease in Patients Admitted to Tertiary Care Hospitals**
Jeroen Geurtsen, Joachim Doua, Luis Martinez-Martinez, Patricia Ibarra de Palacios, Jeff Powis, Matthew Sims, Peter Hermans, Olivier Barraud, Philippe Lanotte, Joshua T. Thaden, Oscar Go, Bart Spiessens, Darren Abbanat, Florian Wagenlehner, Tetsuya Matsumoto, Marc Bonten, Michal Sarnecki, Jan Poolman, on behalf of the BAC0006 Study Group as instructed by the Study Steering Committee.

**Supplementary Material.**

**Supplementary Table S1**. Antibiotic resistance in all patients with IED

**Supplementary Table S2**. Prevalence of O-serotypes based on whole genome sequencing, with a frequency of ≥1%, in all patients with IED

**Supplementary Table S3**. Prevalence of EXPEC9V O-serotypes based on whole genome sequencing, stratified by mortality status, in all patients with IED

**Supplementary Table S4**. Prevalence of EXPEC9V O-serotypes based on whole genome sequencing, stratified by sepsis status, in all patients with IED

**Supplementary Figure S1.** Prevalence (%) of EXPEC9V O-serotypes based on agglutination and whole genome sequencing with cumulative prevalence (%) for MDR IED isolates from patients ≥60 years; full analysis set.

**Supplementary Table S1.** Antibiotic resistance in all patients with IED

| Parameter, n (%) | Number of isolates (*N* = 895)^a^ |
| --- | --- |
| Resistance | |
| Susceptible^b^ | 308 (34.4) |
| MDR^c^ | 179 (20.0) |
| Resistance to single antibiotics | |
| Aminoglycoside | |
| Amikacin | 3 (0.3) |
| Gentamicin | 95 (10.6) |
| Tobramycin | 105 (11.7) |
| Fluoroquinolone | |
| Ciprofloxacin | 240 (26.8) |
| Levofloxacin | 227 (25.4) |
| folate pathway inhibitors | |
| Trimethoprim | 179 (20.0) |
| Trimethoprim/sulfamethoxazole | 261 (29.2) |
| Nitrofurantoin | 3 (0.3) |
| β-lactam | |
| Amoxicillin | 517 (57.8) |
| Amoxicillin/clavulanate | 301 (33.6) |
| Aztreonam | 72 (8.0) |
| Cefepime | 97 (10.8) |
| Cefoxitin | 46 (5.1) |
| Ceftazidime | 96 (10.7) |
| Ceftriaxone | 140 (15.6) |
| Cefuroxime | 185 (20.7) |
| Ertapenem | 6 (0.7) |
| Imipenem | 2 (0.2) |
| Meropenem | 2 (0.2) |
| Piperacillin | 490 (54.7) |
| Piperacillin/tazobactam | 38 (4.2) |
| Temocillin | 71 (7.9) |

^a^895/902 *E. coli* isolates from all patients in the full analysis set underwent antibacterial susceptibility testing.
^b^ Susceptible was based on susceptibility to representative antibiotics in the following five classes of antimicrobial drugs: aminoglycoside, fluoroquinolone, folate pathway inhibitor, nitrofurantoin, and β-lactam. Susceptibility was defined as the absence of resistance to 5/5 antimicrobial drug classes.
^c^MDR was based on resistance to representative antibiotics in the following five classes of antimicrobial drugs: aminoglycoside, fluoroquinolone, folate pathway inhibitor, nitrofurantoin, and β-lactam. MDR was defined as resistance to at least 3/5 antimicrobial drug classes.
Note: susceptibility results are based on EUCAST 2022 breakpoints.
EUCAST, European Committee on Antimicrobial Susceptibility Testing; IED, invasive *E. coli* disease; MDR, multidrug resistant.

**Supplementary Table S2.** Prevalence of O-serotypes based on whole genome sequencing, with a frequency of ≥1%, in all patients with IED

| O-serotype | Number of isolates (*N* = 837) | Prevalence (95% CI)  1.0000 |
| --- | --- | --- |
| O25 | 145 | 0.1732 (0.1482, 0.2006) |
| O2 | 98 | 0.1171 (0.0961, 0.1408) |
| O6 | 78 | 0.0932 (0.0744, 0.1149) |
| O1 | 53 | 0.0633 (0.0478, 0.0820) |
| O15 | 44 | 0.0526 (0.0385, 0.0699) |
| O75 | 42 | 0.0502 (0.0364, 0.0672) |
| O8 | 35 | 0.0418 (0.0293, 0.0577) |
| O16 | 34 | 0.0406 (0.0283, 0.0563) |
| O17_O44_O73_O77_O106 | 34 | 0.0406 (0.0283, 0.0563) |
| O4 | 33 | 0.0394 (0.0273, 0.0549) |
| O18 | 25 | 0.0299 (0.0194, 0.0438) |
| O9 | 23 | 0.0275 (0.0175, 0.0409) |
| O153 | 13 | 0.0155 (0.0083, 0.0264) |
| O13 | 12 | 0.0143 (0.0074, 0.0249) |
| O22 | 10 | 0.0119 (0.0057, 0.0219) |
| O7 | 10 | 0.0119 (0.0057, 0.0219) |
| O101_O162 | 9 | 0.0108 (0.0049, 0.0203) |
| O107_O117 | 9 | 0.0108 (0.0049, 0.0203) |
| O86 | 9 | 0.0108 (0.0049, 0.0203) |

IED, invasive *E. coli* disease.

**Supplementary Table S3.** Prevalence of EXPEC9V O-serotypes based on whole genome sequencing, stratified by mortality status, in all patients with IED

|  | Mortality: Yes | | Mortality: No | |
| --- | --- | --- | --- | --- |
| O-serotype | Number of isolates (*N* = 165) | Prevalence (95% CI) 1.0000 | Number of isolates (*N* = 672) | Prevalence (95% CI)  1.0000 |
| ExPEC9V | 105 | 0.6364 (0.5580, 0.7097) | 447 | 0.6652 (0.6281, 0.7008) |
| O1 | 4 | 0.0242 (0.0066, 0.0609) | 49 | 0.0729 (0.0544, 0.0953) |
| O2 | 13 | 0.0788 (0.0426, 0.1310) | 85 | 0.1265 (0.1023, 0.1540) |
| O4 | 5 | 0.0303 (0.0099, 0.0693) | 28 | 0.0417 (0.0279, 0.0597) |
| O6 | 7 | 0.0424 (0.0172, 0.0855) | 71 | 0.1057 (0.0834, 0.1314) |
| O15 | 10 | 0.0606 (0.0294, 0.1086) | 34 | 0.0506 (0.0353, 0.0700) |
| O16 | 6 | 0.0364 (0.0135, 0.0775) | 28 | 0.0417 (0.0279, 0.0597) |
| O18 | 7 | 0.0424 (0.0172, 0.0855) | 18 | 0.0268 (0.0160, 0.0420) |
| O25 | 41 | 0.2485 (0.1846, 0.3216) | 104 | 0.1548 (0.1282, 0.1844) |
| O75 | 12 | 0.0727 (0.0381, 0.1236) | 30 | 0.0446 (0.0303, 0.0631) |
| Other | 60 | 0.3636 (0.2903, 0.4420) | 225 | 0.3348 (0.2992, 0.3719) |

IED, invasive *E. coli* disease.

**Supplementary Table S4.** Prevalence of EXPEC9V O-serotypes based on whole genome sequencing, stratified by sepsis status, in all patients with IED

|  | Sepsis: Yes | | Sepsis: No | |
| --- | --- | --- | --- | --- |
| O-serotype | Number of isolates  (*N*= 550) | Prevalence (95% CI) 1.0000 | Number of isolates  (*N* = 286) | Prevalence (95% CI) 1.0000 |
| ExPEC9V | 362 | 0.6582 (0.6169, 0.6978) | 190 | 0.6643 (0.6064, 0.7189) |
| O1 | 24 | 0.0436 (0.0282, 0.0642) | 29 | 0.1014 (0.0690, 0.1424) |
| O2 | 67 | 0.1218 (0.0957, 0.1521) | 31 | 0.1084 (0.0748, 0.1503) |
| O4 | 19 | 0.0345 (0.0209, 0.0534) | 14 | 0.0490 (0.0270, 0.0808) |
| O6 | 47 | 0.0855 (0.0635, 0.1120) | 31 | 0.1084 (0.0748, 0.1503) |
| O15 | 28 | 0.0509 (0.0341, 0.0727) | 16 | 0.0559 (0.0323, 0.0893) |
| O16 | 25 | 0.0455 (0.0296, 0.0664) | 9 | 0.0315 (0.0145, 0.0589) |
| O18 | 18 | 0.0327 (0.0195, 0.0512) | 7 | 0.0245 (0.0099, 0.0498) |
| O25 | 105 | 0.1909 (0.1589, 0.2263) | 40 | 0.1399 (0.1018, 0.1855) |
| O75 | 29 | 0.0527 (0.0356, 0.0749) | 13 | 0.0455 (0.0244, 0.0765) |
| Other | 188 | 0.3418 (0.3022, 0.3831) | 96 | 0.3357 (0.2811, 0.3936) |

IED, invasive *E. coli* disease.

| **Supplementary Figure S1.** Prevalence (%) of EXPEC9V O-serotypes based on agglutination and whole genome sequencing with cumulative prevalence (%) for MDR^a^ IED isolates from patients ≥60 years; full analysis set. |
| --- |
| 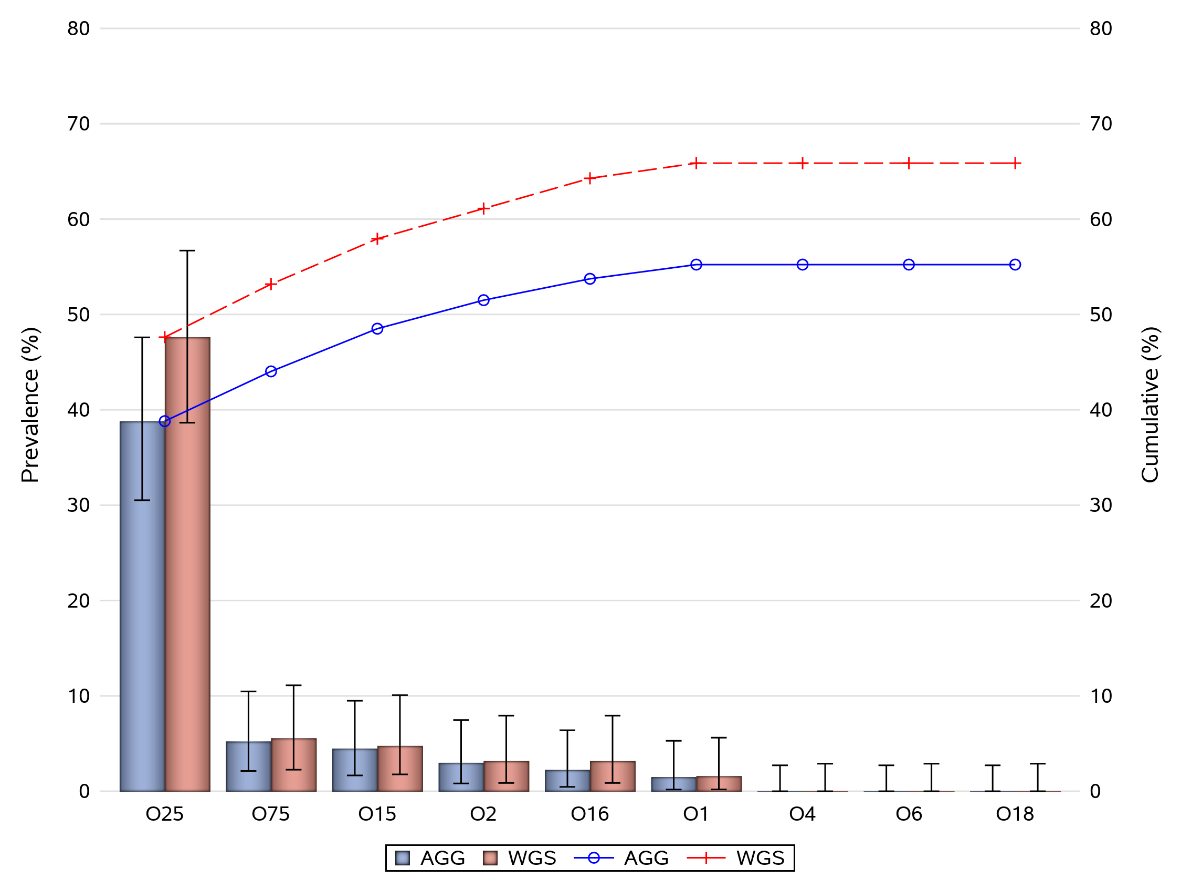 |
| 95% confidence interval based on exact Clopper-Pearson method. ^a^MDR was based on resistance to representative antibiotics in the following five classes of antimicrobial drugs: aminoglycoside, fluoroquinolone, folate pathway inhibitor, nitrofurantoin, and β-lactam. MDR was defined as resistance to at least 3/5 antimicrobial drug classes. Note: susceptibility results are based on EUCAST 2022 breakpoints.  AGG, agglutination; EUCAST, European Committee on Antimicrobial Susceptibility Testing; IED, invasive E. coli disease; MDR, multidrug resistance; WGS, whole genome sequencing. |
